# Knowledge mapping of alström syndrome research: a bibliometric and visualization analysis based on WoS data from 2000 to 2025

**DOI:** 10.3389/fgene.2026.1812729

**Published:** 2026-06-09

**Authors:** Heng Zhang, Hang Fu, Xiaohui Sui, Shangan Si, Yuan Zhang, Kaifeng Li, Mengran Wang, Zhe Song, Yuxin Yang, Ziqi Liu, Guiju Zhang

**Affiliations:** 1 First Clinical Medical College, Shandong University of Traditional Chinese Medicine, Jinan, China; 2 Department of Pediatrics, Affiliated Hospital of Shandong University of Traditional Chinese Medicine, Jinan, China; 3 Department of Geriatrics (Healthcare), Provincial Geriatric Hospital of Traditional Chinese Medicine, Affiliated Hospital of Shandong University of Traditional Chinese Medicine, Jinan, China

**Keywords:** ALMS1, alstrom syndrome, bibliometric analysis, ciliopathy, web of science (WOS)

## Abstract

Alström syndrome (ALMS) is an ultra-rare autosomal recessive disorder caused by mutations in the *ALMS1* gene, leading to a complex spectrum of multi-organ failure, including early-onset sensory loss, obesity, and cardiomyopathy. Despite its clinical significance, a systematic overview of the global research landscape and the intellectual evolution of this field over the past 2 decades remains absent. This study was undertaken to conduct a comprehensive bibliometric and visualization analysis of ALMS research from 2000 to 2025, aiming to identify foundational contributions, evaluate international collaboration patterns, and pinpoint emerging research frontiers. Utilizing data from the Web of Science Core Collection, 345 relevant English-language articles and reviews were analyzed using VOSviewer, CiteSpace, and the R-bibliometrix package. Our findings revealed an exponential growth phase in publications post-2020, with developed nations dominating the research output and Jackson Laboratory serving as a critical international hub. Prolific contributors such as Jan D. Marshall and Pietro Maffei have established dense collaboration networks that facilitate the transition of ALMS research from early genetic characterization to contemporary precision medicine. Keyword and co-citation analysis further highlight a thematic shift toward “ciliopathy” contexts, with recent hotspots focusing on “whole exome sequencing” and the management of “cardiomyopathy”. These results imply that while our understanding of ALMS pathogenesis is maturing, significant challenges in phenotypic heterogeneity and the lack of targeted therapies persist. Future research should prioritize interdisciplinary resource integration and the application of advanced genomics to optimize clinical management and patient outcomes in this complex rare disease.

## 1 Introduction

Alström syndrome (ALMS) is an exceedingly rare autosomal recessive disorder distinguished by its multi-system involvement (Dauleh et al., 2025). This condition manifests through early-onset vision and hearing impairment, obesity, insulin resistance, cardiomyopathy, as well as liver and kidney dysfunction (Zmysłowska-Polakowska et al., 2025a). Clinical symptoms typically present in infancy, with both the severity and the number of affected organ systems escalating over time (Tahani et al., 2020). The etiology of ALMS is attributed to mutations in the *ALMS1* gene (Zmysłowska-Polakowska et al., 2025b), with an estimated prevalence ranging from 1/1,000,000 to 1/100,000. To date, approximately 1,200 cases of Alström syndrome have been recorded globally (Hanaki et al., 2024). The clinical features are intricate and tend to deteriorate progressively, often resulting in severe complications and premature mortality (Wanninayake et al., 2025).

Although several studies have illuminated the role of the *ALMS1* protein in ciliary function (Hearn et al., 2005; Bea-Mascato et al., 2025), metabolic regulation, and the cell cycle (Álvarez-Satta et al., 2015; Zhang et al., 2025), the overarching pathophysiological mechanisms remain elusive, and there is currently an absence of targeted disease-modifying therapies. Over the last 2 decades, advancements in genetics, molecular biology, and clinical medicine have significantly enhanced our comprehension of this syndrome, notably in terms of genotype-phenotype correlations, multi-organ management, and the importance of early intervention (Qi et al., 2025). Nonetheless, owing to the rarity of the disease, the disparate nature of reported cases, and the pronounced phenotypic heterogeneity, considerable gaps persist in research. These gaps include a comprehensive understanding of the underlying pathogenesis, the establishment of standardized diagnostic and treatment protocols, and the development of effective therapeutic strategies (Sinha et al., 2025; Hu et al., 2024).

Bibliometric analysis, serving as a quantitative methodology for evaluating scientific output, has emerged as a pivotal tool for elucidating research trends, identifying collaborative academic networks, and forecasting the trajectories of disciplinary development (Niu et al., 2025). Prior bibliometric investigations within the realm of rare diseases such as those concerning Huntington’s disease (Tang et al., 2025) and cystic fibrosis (Karcioglu et al., 2025) have adeptly delineated the knowledge structure, key contributors, and evolutionary pathways within these domains, thereby establishing a foundation for resource integration and research strategizing. Nevertheless, a systematic bibliometric study dedicated to Alström syndrome that thoroughly maps its research landscape, international collaboration patterns, and the evolution of knowledge remains conspicuously absent. This lacuna hampers our comprehensive understanding of significant contributions, research focal points, and translational hurdles within the field.

Despite the considerable body of literature that has amassed since the syndrome was first characterized, these disparate findings have not been systematically synthesized or visually represented. Consequently, this study endeavors to undertake a bibliometric analysis of literature pertaining to Alström syndrome from 2000 to 2025, with the objective of illustrating the research panorama in this area. By scrutinizing publication trends, identifying core authors, mapping institutional collaboration networks, and analyzing keyword co-occurrence and clustering, the aims of this study are threefold: (1) to pinpoint foundational contributions and emerging Frontier themes; (2) to evaluate the status of international scientific collaboration; and (3) to uncover current translational challenges and prospective opportunities. The anticipated outcomes of this research are designed to furnish structured references for researchers, clinical practitioners, and policymakers, thereby facilitating the optimization of research resource allocation and fostering innovation in the investigation of mechanisms and clinical management of Alström syndrome.

## 2 Materials and methods

### 2.1 Search strategy and data collection

The data for this study were meticulously sourced from the esteemed Web of Science Core Collection (WoSCC). The search was conducted on 28 December 2025, encompassing the period from 1 January 2000, to 31 December 2025. To enhance data clarity and consistency, the language of the literature was restricted to English, and the document types were limited to “Article” and “Review.”

**FIGURE 1:**
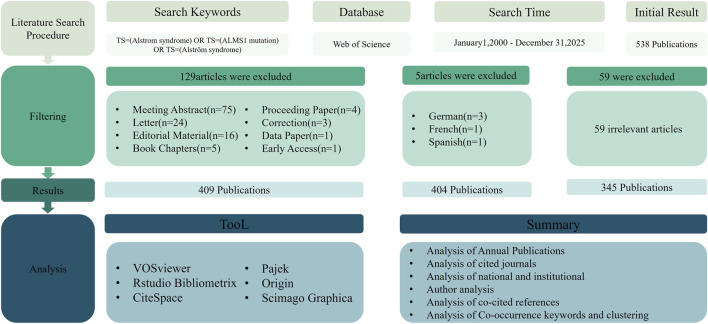
Flow chart of this study.

The search strategy utilized Boolean logic operators to thoroughly encompass research pertinent to Alström syndrome, employing specific search terms: TS=(Alstrom syndrome) OR TS=(ALMS1 mutation) OR TS=(Alström syndrome). Preliminary search results were subsequently exported in the “Full Record and Cited References” format and saved as plain text files. To ensure compatibility with subsequent analytical software, the downloaded files were renamed in accordance with standardized protocols, as depicted in Figure 1. The data selection process strictly adhered to the PRISMA guidelines (Page et al., 2021). Given that all data were acquired from publicly accessible databases, this study did not necessitate ethical approval.

Regarding exclusion, a total of 129 records were removed based on document types, including meeting abstracts (n = 75), letters (n = 24), editorial materials (n = 16), book chapters (n = 5), proceeding papers (n = 4), corrections (n = 3), data papers (n = 1), and early access articles (n = 1). To facilitate standardized data processing, non-English publications were excluded, including articles in German (n = 3), French (n = 1), and Spanish (n = 1). Finally, after a preliminary manual review of titles and abstracts, 59 irrelevant articles were removed. The initial search yielded 538 publications, and after applying these criteria, a total of 345 eligible publications remained for the final bibliometric analysis.

Before conducting the visualization, data preprocessing was performed to ensure accuracy. First, we utilized the ‘Thesaurus’ function in VOSviewer and alias files in CiteSpace to merge synonymous keywords (e.g., ‘Alström syndrome’ and ‘Alstrom syndrome’ were unified). Second, we manually standardized author names and institutional affiliations to resolve spelling variations. Third, any irrelevant records identified during the manual screening phase were removed from the final dataset used for mapping.

### 2.2 Statistical analysis

For the analysis and visualization of the data, we employed VOSviewer (version 1.6.20), CiteSpace (version 6.1.R3), and the “bibliometrix” package in R (version 4.3.3). VOSviewer was chosen for its adeptness in mapping bibliometric indicators and visualizing intricate collaboration and relationship networks within academic disciplines (van Eck and Waltman, 2010). Its capabilities in managing large datasets and vividly depicting keyword co-occurrence networks render it particularly effective for discerning emerging research trends and identifying influential publications. In contrast, CiteSpace is extensively utilized for its robust functionality in detecting keyword bursts and visualizing temporal trends (Chen, 2004). Its advanced network pruning methodology is advantageous for generating succinct visualizations that underscore significant shifts in research focus over time (Zhong et al., 2022). The integration of these analytical tools not only enhances the rigor of our analysis but also facilitates a comprehensive exploration of treatment trends in ALMS.

## 3 Results

### 3.1 Analysis of annual publications and trends

**FIGURE 2:**
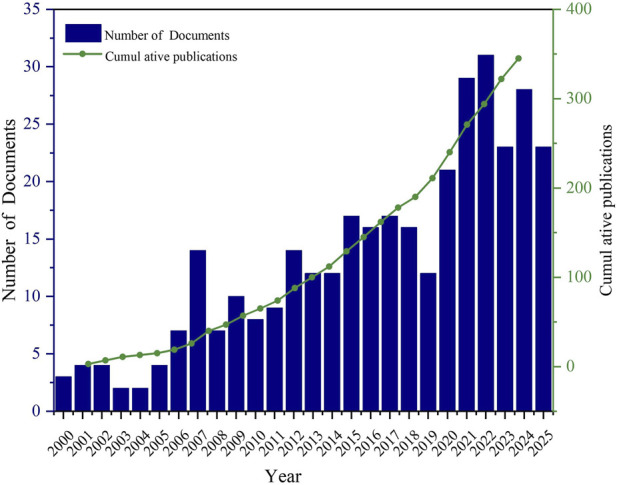
The annual publication quantity and cumulative publication quantity of articles on Alström syndrome.

Figure 2 depicted consistent upward trajectory in publications over the past 25 years, with a particularly pronounced acceleration in recent years. This temporal progression can be discerned in three distinct phases, which are statistically justified by changes in the growth rate of cumulative publications (Figure 2) and significant academic milestones. The initial phase, spanning from 2000 to approximately 2005, represents a foundational period characterized by low output; annual publications lingered in the single digits, exhibiting minimal linear growth. The cumulative count gradually ascended from around three to 19, suggesting that this field was largely a niche domain focused on foundational discoveries, preliminary proofs of concept, and the establishment of fundamental methodological frameworks.A pivotal turning point occurred around 2006, ushering in a second phase distinguished by accelerated near-linear growth that persisted until approximately 2019. During this interval, annual publication counts began to consistently reach double digits, with cumulative totals surging from about 26 to 190. This period marked a significant expansion in research focus, reflecting a burgeoning interest in the complexities of the syndrome. The most striking aspect of the data is the pronounced exponential growth phase that commenced around 2020. Although the annual publication count stabilized on a high plateau, the cumulative curve demonstrated its steepest incline, rising from approximately 190 to an estimated 345 by 2025. This phase signifies the most rapid accumulation of knowledge throughout the entire timeline, underscoring a dynamic shift in scholarly attention towards ALMS.

### 3.2 Analysis of cited journals

Table 1 presents a ranking of the top 10 journals within this research domain, unveiling several noteworthy observations. The “Orphanet Journal of Rare Diseases” holds the premier position throughout the study period, having published 12 articles and garnered a total of 202 citations. In this field, there exists a total of 206 journals, collectively accruing an impressive citation count of 9,885. Notably, the combined citations of the top 10 journals amount to 1,637, which constitutes approximately 16.56% of the overall citation total. This statistic underscores the substantial impact and prominence of these journals within the broader scholarly landscape, highlighting their significance in advancing research in the domain of ALMS. Of particular significance is the “Journal of Clinical Endocrinology & Metabolism,” which distinguishes itself among the listed publications with 484 citations and the highest impact factor. This highlights its pivotal role and influence within the field, underscoring its status as a leading platform for disseminating research related to ALMS and its associated domains.

**TABLE 1:** The top 10 journals publishing research on ALMS.

Rank	Source	Documents	Citations	Total link strength	JCR	Impact factor
1	*Orphanet Journal of Rare Diseases*	12	202	56	2	3.5
2	*American Journal of Medical Genetics Part A*	10	179	49	3	1.7
3	*Plos One*	7	290	37	3	2.6
4	*Human Mutation*	7	21	63	2	3.7
5	*Journal of Clinical Endocrinology & Metabolism*	6	484	29	2	5.1
6	*Clinical Genetics*	6	147	17	3	2.3
7	*Ophthalmic Genetics*	6	145	14	4	1
8	*European Journal of Medical Genetics*	6	96	35	4	1.7
9	*Frontiers in Genetics*	6	36	32	3	2.8
10	*Gene*	5	37	20	3	2.4

**FIGURE 3:**
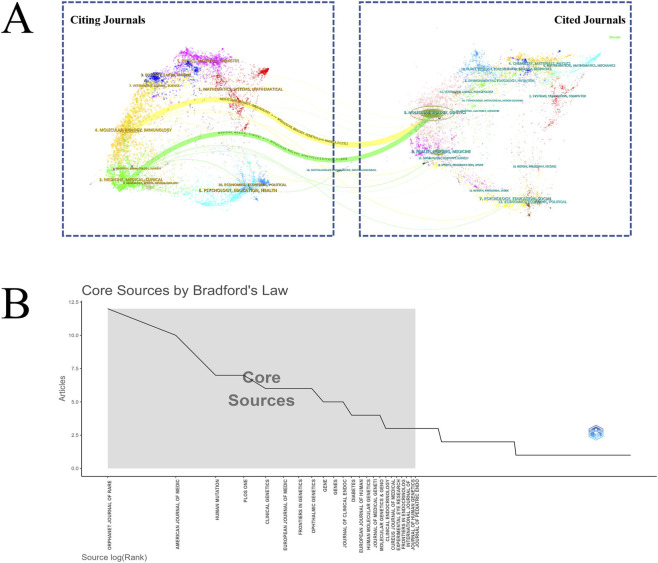
**(A)** Bipartite overlay diagram. **(B)** Distribution map of core journals.

The analysis of the bipartite overlay of journals offers insights into the development of the discipline and tracks advancements at the scientific Frontier (Yang et al., 2024). Figure 3A illustrates a bipartite overlay of journals within the field, with citing journals positioned on the left and cited journals on the right. The colored curves connecting both sides represent the primary citation flows. Notably, the yellow line indicates that articles from the domain of Molecular/Biology/Genetics are predominantly cited by those related to Molecular/Biology/Immunology. Additionally, the green pathways highlight that citing journals primarily focus on Medicine/Medical/Clinical topics, while the cited journals are largely drawn from Molecular/Biology/Genetics. This visualization effectively captures the interdisciplinary knowledge base and dissemination pathways prevalent in the research field.

Furthermore, as shown in Figure 3B, Bradford’s Law was employed to identify the core journals in the field, ultimately identifying 23 core journals (Li et al., 2025). The figure plots the number of articles against the logarithm of journal rankings, with the gray shaded area on the left distinctly marking the core journal zone. This representation quantitatively and visually elucidates the core-periphery structure inherent in AMLS research publication patterns.

### 3.3 National and institutional analysis

**FIGURE 4:**
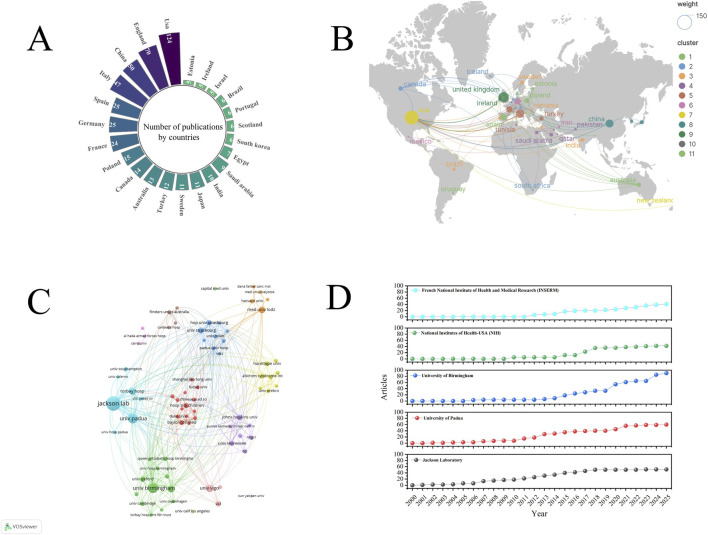
**(A)** Statistics of the number of publications issued by countries. **(B)** Geographic map of national collaboration. **(C)** Institution co- occurrence network diagram. **(D)** Institution stacked line chart.

A total of 65 countries have contributed to the body of literature pertaining to the ALMS field. As illustrated in Figure 4A and detailed in Table 2, the top ten countries ranked by publication count are as follows: the United States (124), the United Kingdom (70), China (50), Italy (47), Spain (25), Germany (25), France (24), Poland (15), Canada (14), and Australia (13).

**TABLE 2:** The top 10 most productive countries.

Rank	Country	Documents	Citations	Total link strength
1	United States of America	124	6247	2907
2	England	69	3281	2311
3	Italy	46	2833	2062
4	China	41	1244	784
5	Germany	25	1743	873
6	Spain	25	719	743
7	France	24	1022	865
8	Poland	15	289	308
9	Canada	14	1795	458
10	Australia	13	810	541

Notably, the United States emerges as the leader with 124 publications, representing the highest proportion among the top ten contributors. The United Kingdom follows closely with 70 publications. Among these leading nations, developed countries account for a substantial 90% of the total output, while developing nations contribute the remaining 10%. This finding underscores the significant contributions made by developed nations in this research domain, highlighting their preeminent role and reflecting the increasing global focus on ALMS research.

Moreover, this trend emphasizes the growing strength of international collaboration and knowledge exchange within the field, indicating a dynamic and interconnected research environment that fosters advancements in understanding and addressing the complexities of Alström syndrome.

As depicted in Figure 4B, the global collaboration network within this field exhibits a pronounced core-periphery structure, with the United States positioned as the central and most intricately connected hub. Its extensive and formidable links extend across North America, Europe, and Asia, showcasing the highest intensity and breadth of international collaboration.

Conversely, although the United Kingdom leads in publication volume, its role and connectivity within this particular collaboration network appear less central and extensive when compared to that of the United States. The visualization reveals distinct regional collaboration clusters, each marked by varying colors, with a particularly strong interconnected sub-cluster among European nations. Additional collaborative groups are also identifiable in Asia and the Middle East.

The “weight” of these connections is represented by the thickness of the lines, which visually emphasizes the disparities in collaboration intensity between countries, particularly accentuating the connections stemming from the United States. This network structure underscores that robust international collaboration is predominantly concentrated around a select few developed nations, and the high-intensity cooperation among leading research powers is widely acknowledged as a critical driver of research excellence and innovation.

As illustrated in Figure 4C, Jackson Laboratory (USA) stands out as a central and intricately connected hub within the international collaboration network, underscoring its robust and extensive partnerships. It serves as a primary connector among various global research clusters.

In Europe and other regions, several institutions exhibit significant international linkages. The University of Southampton (UK) and the University of Salerno (Italy) emerge as vital nodes within their respective collaboration clusters. Furthermore, Cairo University (Egypt) and the Armed Forces Hospital (Saudi Arabia) demonstrate active cross-regional research partnerships, contributing to the diversity of the collaboration landscape.

Overall, the visualization reveals that global research collaboration is predominantly driven by leading North American institutions, which form the core of the network. From this central hub, dense connections extend outward, establishing extensive cooperative relationships with academic and medical centers worldwide, including those in Europe, the Middle East, and beyond. This interconnectedness not only enhances the flow of knowledge but also fosters a collaborative environment that is essential for advancing research in the field.

Figure 4D illustrates the annual productivity of five leading institutions from 2000 to 2025, all exhibiting significant global growth, albeit with varying trajectories. The National Institutes of Health (NIH) has shown steady growth for most of this period, entering a peak output plateau post-2020. Notably, the University of Birmingham experienced the most pronounced increase, with its publication rate sharply rising around 2015, making it the highest-producing institution in the group by 2021. This indicates a significant strategic expansion of its research capacity. Other institutions, such as the French National Institute of Health and Medical Research (INSERM) and the University of Padua, displayed stable, incremental growth. Although Jackson Laboratory is smaller in scale, it has maintained a consistent upward trend in its specialized field. The output peaks observed around 2021 may be associated with heightened research activity during the COVID-19 pandemic, followed by a period of stability or moderate growth in subsequent years, reflecting the evolution of the research landscape in the post-pandemic era.

### 3.4 Author analysis

**FIGURE 5:**
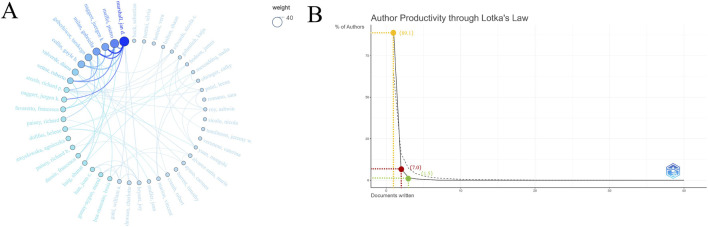
**(A)** Author chord diagram. **(B)** Author productivity through Lotka’s law.

Table 3 and Figure 5A present an analysis of publication volume, citation counts, and collaboration intensity among the top ten authors. In the figure, the varying sizes of nodes and the thickness of connecting lines represent the magnitude and strength of collaborative relationships, while the central cluster signifies particularly robust and frequent partnerships. Noteworthy contributions are identified, with Jan D. Marshall leading with 32 publications and Pietro Maffei following closely with 26, establishing them as pivotal figures in the field. Their substantial publication outputs highlight not only their academic productivity but also the significance of their collaborative networks in advancing novel insights within AMLS research. Additionally, Figure 5B reveals that 89.1% of authors have published only one paper, with a notable decline in the number of corresponding authors as the number of publications increases.

**TABLE 3:** The top 10 most productive authors.

Rank	Author	Documents	Citations	Total link strength
1	Jan D. Marshall	32	1298	1072
2	Pietro Maffei	26	1212	1060
3	Jurgen K. Naggert	19	1044	751
4	Gabriella Milan	19	718	853
5	Gayle B. Collin	17	929	652
6	Tarekegn Geberhiwot	17	302	448
7	Diana Valverde	14	266	424
8	Roberto Vettor	12	505	575
9	Francesca Favaretto	9	424	559
10	Richard P. Steeds	9	123	218

### 3.5 Analysis of co-cited references

Co-citation refers to the occurrence of two or more articles being cited together by other articles (Song et al., 2022). Table 4 lists the ten most co-cited references. The most cited article is “New Alström Syndrome Phenotypes Based on the Evaluation of 182 Cases,” published in 2005 in Arch Intern Med.

**TABLE 4:** The top 10 most co-cited references.

Rank	First author	Title	Journal	Year	Citations
1	Jan D Marshall	New Alström syndrome phenotypes based on the evaluation of 182 cases	Arch Intern Med	2005	145
2	Gayle B Collin	Mutations in ALMS1 cause obesity, type 2 diabetes and neurosensory degeneration in Alström syndrome	Nat Genet	2002	140
3	Jan D Marshall	Alström syndrome: Genetics and clinical overview	Curr genomics	2011	123
4	Tom Hearn	Mutation of ALMS1, a large gene with a tandem repeat encoding 47 amino acids, causes Alström syndrome	Nat Genet	2002	122
5	Jan D Marshall	Alström syndrome	Eur J Hum Genet	2007	121
6	Tom Hearn	Subcellular localization of ALMS1 supports involvement of centrosome and basal body dysfunction in the pathogenesis of obesity, insulin resistance, and type 2 diabetes	Diabetes	2005	92
7	Jan D Marshall	Alström syndrome: Mutation spectrum of ALMS1	Hum Mutat	2015	87
8	Jan D Marshall	Spectrum of ALMS1 variants and evaluation of genotype-phenotype correlations in Alström syndrome	Hum Mutat	2007	86
9	Guochun Li	A role for Alström syndrome protein, alms1, in kidney ciliogenesis and cellular quiescence	PLoS Genet	2007	77
10	G B Collin	Alms1-disrupted mice recapitulate human Alström syndrome	Hum Mol Genet	2005	72

**FIGURE 6:**
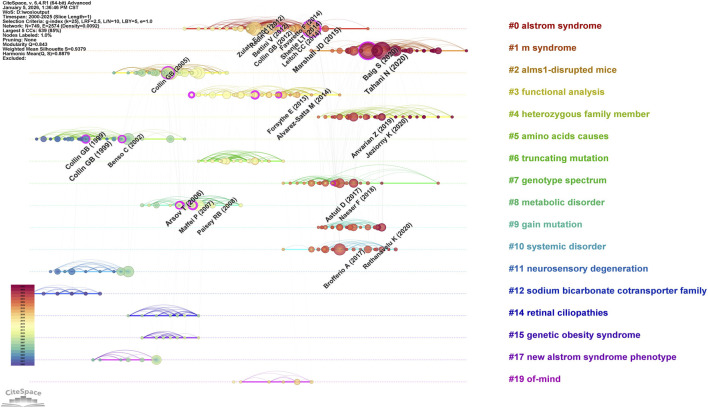
Timeline of references.

Figure 6 presents a timeline view of co-cited references from 2002 to 2025. Nodes connected by curves indicate simultaneous citations of entries. The color of the citation rings represents the distribution of citation times, with each cluster’s timeline displayed chronologically. Each cluster is labeled with abstract terms from the cited articles. The analysis using CiteSpace reveals the research development and evolutionary span of each cluster. Cluster labels include “alstrom syndrome,” “m syndrome,” “*alms1*-disrupted mice,” “functional analysis,” “heterozygous family member,” “amino acids causes,” “truncating mutation,” “genotype spectrum,” “metabolic disorder,” “gain mutation,” “systemic disorder,” “neurosensory degeneration,” “sodium bicarbonate cotransporter family,” “retinal ciliopathies,” “genetic obesity syndrome,” “new alstrom syndrome phenotype,”and “of-mind.”

### 3.6 Analysis of keywords

Keyword clustering involves organizing similar thematic keywords into distinct groups, while co-occurrence analysis reveals the relationships between various topics within the same discipline (Chen et al., 2012). This process is akin to the clustering of references, where labels for keyword clusters are derived from the keywords found in the titles of relevant cited articles. By employing keyword co-occurrence and clustering analysis, researchers can gain insights into the structure of scientific knowledge networks, ultimately helping to identify research hotspots within major professional fields. This approach enhances our understanding of emerging trends and the interconnectedness of different topics in the research landscape.

**FIGURE 7:**
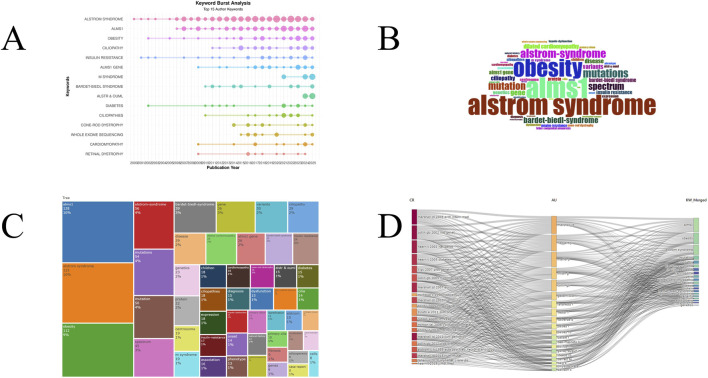
**(A)** Keyword burst graph. **(B)** Keyword word cloud. **(C)** Keyword tree diagram. **(D)** Sankey diagram.

Keyword co-occurrence and clustering analysis serve as more than a statistical tally of term frequencies, acting instead as a profound mapping of the scientific knowledge network within the field. As illustrated in Figure 7, these thematic clusters directly reflect the research community’s real-time response to clinical challenges and technological advancements. Within the burst intensity analysis shown in Figure 7A, the keyword “Alström syndrome” exhibits a sustained and prominent burst from 2000 to 2025, establishing its foundational role as the root of the entire disciplinary network. Following closely is the gene-related term “*ALMS1*,” whose burst period between 2008 and 2012 aligns precisely with the early functional characterization of the causative gene and initial genotype-phenotype association studies (Taşdemir et al., 2013; Kocova et al., 2011). This evolution marks a critical transition in the research paradigm from purely clinical descriptions to molecular mechanism exploration.

Over time, the thematic network demonstrates a clear logical expansion. The emergence of broader conceptual keywords such as “ciliopathy” between 2010 and 2016 was a milestone, indicating that researchers began to frame ALMS within the larger biological context of ciliary signaling defects. This shift in perspective allowed the scientific community to leverage mechanistic insights from related disorders, such as Bardet-Biedl syndrome, to achieve a more profound understanding of the regulatory role of the *ALMS1* protein within the centrosomal-ciliary complex.

The most significant technological leap after 2020 is reflected in the explosive growth of “whole exome sequencing” (WES). This trend precisely addresses a major clinical challenge, namely, the difficulty of distinguishing ALMS from other phenotypically overlapping rare diseases during early infancy. The application of high-throughput genomic technologies has not only significantly improved diagnostic precision but has also driven the discovery of population-specific mutational spectra, such as the unique splice-site variants revealed by recent studies of large Chinese cohorts.

Concurrently, the sustained focus on phenotype-specific keywords like “cardiomyopathy” and “retinal dystrophy” directly maps the most urgent survival challenges faced by patients. Since cardiomyopathy remains the primary cause of mortality during infancy in ALMS, the enduring intensity of this cluster reflects a shift in research priorities from identifying genetic codes to managing progressive multi-organ failure. Figures 7B,C further confirm the unity of the gene-disease relationship, while the Sankey diagram in Figure 7D clearly illustrates the flow of knowledge, clarifying how core scholars like Jan D. Marshall have guided research from early genetic foundations toward contemporary precision medicine. Current research frontiers are dedicated to utilizing advanced omics technologies to address metabolic disturbances and lethal complications, moving the field from a descriptive stage to one of targeted intervention.

## 4 Discussion

### 4.1 General information and knowledge base

This bibliometric analysis provides a comprehensive longitudinal overview of the research landscape surrounding ALMS from 2000 to 2025. The results delineate a consistent upward trajectory in scientific output, which can be strategically divided into three developmental phases based on statistical inflection points observed in Figure 2 and significant academic milestones.

Phase I (2000–2005) represents the “Etiology Foundation Period.” Following the landmark identification of the *ALMS1* gene in 2002, research was initially confined to a niche domain focused on defining molecular foundations. During this stage, the annual publication volume remained in the single digits, reflecting a period of nascent discovery where the scientific community was primarily concerned with early genetic characterization. Phase II (2006–2019) marks the “Steady Development Phase.” During this interval, the cumulative curve shows a linear increase, indicating that ALMS research moved from an isolated genetic discovery to a more established field of study. This phase saw the emergence of the “ciliopathy” concept, which unified ALMS with other disorders involving ciliary dysfunction. The focus expanded from pure genetics to comprehensive phenotypic documentation across diverse cohorts. Phase III (post-2020): The exponential surge in this phase is a direct consequence of the “Genomic Revolution.” The widespread accessibility of WES has lowered the diagnostic threshold, allowing for the identification of atypical or “pauci-symptomatic” cases that previously eluded detection. This trend suggests that the field is moving from descriptive genetics to a more proactive, screening-oriented paradigm.

### 4.2 Critical insights into mutational analysis: addressing the Translational Paradox

A deeper biological interpretation of the genetic data is a critical requirement for advancing ALMS research. While variants are highly concentrated in exons 8, 10, and 16, this clustering highlights a significant “Translational Paradox”: despite high-resolution mapping of DNA mutations, our understanding of how these mutations translate into vast phenotypic diversity remains incomplete.

Our analysis confirms that most pathogenic variants lead to protein loss via nonsense-mediated decay (NMD). However, the high inter-individual variability in cardiomyopathy onset, even among siblings with identical mutations, critically implies that *ALMS1* does not operate in a vacuum. A major “Research Gap” exists in identifying the genetic modifiers and epigenetic regulators that influence disease severity. Future studies must pivot from simple variant reporting to functional transcriptomics to determine why certain truncating mutations escape NMD, potentially leading to partially functional proteins and milder phenotypes. Furthermore, the rise of population-specific studies, such as the analysis of 127 Chinese patients (Huang et al., 2025), challenges the Euro-centric historical bias of rare disease databases. These findings imply that “mutational hotspots” are geographically dependent, necessitating the development of localized screening panels in regions where founder effects are prevalent.

### 4.3 Global collaboration and the epidemiological re-evaluation

The historical dominance of the USA and UK is attributed to their early establishment of centralized patient registries and specialized centers of excellence like the Jackson Laboratory. This model has provided the backbone for ALMS research for over 2 decades. However, the recent rise of China and other Asian nations signals a shift from a “Centralized Resource Model” to a “Global Network Model,” which is essential for diversifying the genetic pool.

The evolution of prevalence estimates is not an indication of increasing incidence but a critical correction of historical underdiagnosis. This epidemiological shift suggests that as diagnostic capabilities improve in developing nations, the global documented patient population will continue to expand. This underscores the urgent need for international registries to pool data from isolated cohorts, which is essential for conducting statistically significant clinical trials in rare disease populations, a major current bottleneck in therapeutic development.

### 4.4 Future frontiers: from descriptive mapping to disease modification

Looking forward, ALMS research is poised to enter a new era of targeted intervention, yet a major future gap remains the critical lack of disease-modifying therapies beyond symptomatic management. In the realm of molecular therapeutics, the high proportion of nonsense mutations identified in this study identifies a clear strategic target for pharmacological stop-codon read-through agents, suggesting that future research should prioritize small-molecule screenings that can bypass premature termination codons. Furthermore, the transition from DNA-centric to protein-centric studies will be vital for functional proteomics, as elucidating how the *ALMS1* protein interacts within the centrosomal-ciliary complex to regulate insulin signaling and adipogenesis is the missing link in treating the syndrome’s metabolic morbidity. Finally, as pediatric survival improves due to better multidisciplinary care, the field must address the neglected research area of adult-onset complications to close the geriatric research gap. Establishing interdisciplinary care models that merge endocrinology, cardiology, and nephrology remains the gold standard, effectively shifting the focus from survival to long-term quality of life.

## 5 Limitations

This study has several limitations. First, the data were obtained solely from the Web of Science Core Collection. While WoSCC provides the most comprehensive citation metadata for bibliometric tools, it may not capture all relevant publications indexed in other databases like PubMed or Google Scholar, potentially leading to selection bias. Second, restricting the literature to English articles and reviews may exclude influential non-English works, thus limiting the global perspective on the topic. Third, bibliometric methods are inherently quantitative and may not capture qualitative nuances, such as clinical trial outcomes or patient-specific factors. Lastly, projections about publications through 2025 are based on extrapolation and may be influenced by unforeseen trends. Despite these limitations, this analysis provides a solid foundation for understanding the dynamics of ALMS research.

## 6 Conclusion

In summary, this bibliometric analysis delineates the evolution of ALMS research from 2000 to 2025, highlighting a notable surge in publications driven by gene discoveries and international collaboration. The USA leads in output, with key authors like Jan D. Marshall and Pietro Maffei playing significant roles in the field. Research has transitioned from foundational descriptions to precision medicine, with emerging hotspots such as whole exome sequencing and cardiomyopathy management. However, challenges such as phenotypic heterogeneity and the lack of targeted therapies remain significant hurdles for future research and clinical application.
